# The Potential Inhibitory Role of Acetyl-L-Carnitine on Proliferation, Migration, and Gene Expression in HepG2 and HT29 Human Adenocarcinoma Cell Lines

**DOI:** 10.3390/cimb45030155

**Published:** 2023-03-14

**Authors:** Sarah Albogami

**Affiliations:** Department of Biotechnology, College of Science, Taif University, P.O. Box 11099, Taif 21944, Saudi Arabia; dr.sarah@tu.edu.sa

**Keywords:** acetyl-L-carnitine, matrix metallopeptidase 9, vascular endothelial growth factor, human liver adenocarcinoma cell line, human colorectal adenocarcinoma cell line

## Abstract

Malignancies of the liver and colon are the most prevalent forms of digestive system cancer globally. Chemotherapy, one of the most significant treatments, has severe side effects. Chemoprevention using natural or synthetic medications can potentially reduce cancer severity. Acetyl-L-carnitine (ALC) is an acetylated derivative of carnitine essential for intermediate metabolism in most tissues. This study aimed to investigate the effects of ALC on the proliferation, migration, and gene expression of human liver (HepG2) and colorectal (HT29) adenocarcinoma cell lines. The cell viability and half maximal inhibitory concentration of both cancer cell lines were determined using the 3-(4, 5-dimethylthiazol-2-yl)-2, 5-diphenyltetrazolium bromide (MTT) assay. Wound healing after treatment was assessed using a migration assay. Morphological changes were imaged using brightfield and fluorescence microscopy. Post treatment, apoptotic DNA was detected using a DNA fragmentation assay. The relative mRNA expressions of matrix metallopeptidase 9 (MMP9) and vascular endothelial growth factor (VEGF) were evaluated using RT-PCR. The results showed that ALC treatment affects the wound-healing ability of HepG2 and HT29 cell lines. Changes in nuclear morphology were detected under fluorescent microscopy. ALC also downregulates the expression levels of MMP9 and VEGF in HepG2 and HT29 cell lines. Our results indicate that the anticancer action of ALC is likely mediated by a decrease in adhesion, migration, and invasion.

## 1. Introduction

Malignancies of the liver and colon are the most prevalent forms of digestive system cancer globally [1]. Liver cancer is the third most common type of cancer in males and eighth most common in females globally. It is the largest cause of mortality worldwide, and more than 800,000 new cases and 700,000 fatalities are reported annually [2,3,4,5]. Risk factors for liver cancer include chronic hepatitis B virus infection, high alcohol use, obesity, diabetes, and smoking [6,7,8]. Globally, colorectal cancer ranks fourth among males and third among females [9]; obesity, a diet lacking fruits and vegetables, a sedentary lifestyle, and smoking are risk factors [10,11,12]. As an imbalanced diet and altered cellular metabolism are important risk factors for the advancement of both liver and colorectal cancers, dietary adjustments are the first-line treatment [13]. A significant foundation for cancer chemoprevention has been the astonishing number of animal studies demonstrating that a range of chemical substances can prevent cancer [14]. In the pursuit of more effective inhibitors, both synthetic and natural substances are being studied [15]. Carnitine is a hydrophilic substance that plays a crucial function in the transport of long-chain fatty acids for beta-oxidation within mitochondria [16,17,18]. Carnitine is a powerful antioxidant and, given that it absorbs active oxygen species in tissues, carnitine has potential anticancer characteristics [19,20]. Carnitine—is hypothesized to cause a boost in cell respiration and apoptosis, along with a decrease in cell proliferation and inflammation in cancer cells through a variety of pathways [21,22,23]. The influence of carnitine on colon tumor progression has been studied in vivo, utilizing two experimental mouse models of colon cancer. One was an azoxymethane treatment model of carcinogen-induced colorectal cancer, whereas the other was a genetically generated model. These in vivo studies revealed that treatment with carnitine substantially elevated levels of carnitine and acylcarnitine in tissues. In azoxymethane-treated animals carnitine reduced the formation of premalignant lesions, while in a genetically induced model carnitine did not demonstrate tumor-protective properties [24]. Using mice, carnitine palmitoyltransferase I and II activity and cachectic cancer expression in the liver were evaluated in relation to L-carnitine supplementation [24]. The mRNA expression level and activity of liver carnitine palmito-yltransferase I and II, as well as serum levels of carnitine and acetyl-carnitine, were significantly lowered, which is associated with substantial elevations in serum concentrations of interleukin-6 (IL-6) and tumor necrosis factor-alpha (TNF) [24].

However, several forms of carnitine exist, including L-carnitine (LC), acetyl-L-carnitine (ALC), and propionyl-L-carnitine (PLC) [25]. Among these, ALC has received remarkable attention recently due to its various therapeutic properties. ALC is an acetylated derivative of L-carnitine produced by carnitine acetyltransferase with a high degree of bioavailability [26]. Numerous biological functions of ALC are induced by the metabolic effects of its acetyl and carnitine components, which are essential for a variety of intracellular and metabolic processes, including fatty acid transport into mitochondria, the stability of cell membranes, and a decrease in serum lipid concentrations, and its acetyl group can sustain cetyl-CoA levels [27,28]. ALC participates in the translocation of acetyl units across the mitochondrial membranes in both anabolic and catabolic pathways [29], and furthermore it is a common free radical scavenger and a regulator of energy metabolism and metabolic processes [30]. ALC also exhibits anti-apoptotic and anti-inflammatory properties [31,32,33,34,35] in addition to its stabilizing action on the mitochondrial membrane [36]. The clinical application of ALC has been demonstrated to have excellent outcomes in a range of diseases [22]. It has been proposed as a powerful, inexpensive, and safe alternative treatment for patients with cirrhosis [37] It functions at several levels to cure diabetic polyneuropathy type 1 [38], and may also restore equilibrium in diseases causing neuronal ceroid lipofuscinoses [39]. ALC and alpha-lipoic acid have been shown to enhance mitochondrial energy metabolism and reduce oxidative stress, resulting in enhanced memory in old rats [40,41]. Elmirini et al. (2015) demonstrated that ALC might have anticancer effects against colon cancer in vitro [42]; ALC was investigated at a molecular level to see if it functions as a “angiopreventive” substance. Previous in vitro and in vivo studies revealed that ALC inhibits inflammatory angiogenesis by decreasing triggered endothelial cell and macrophage infiltration; on a molecular level, Elmirini et al. demonstrated that ALC inhibits the vascular endothelial growth factor (VEGF), vascular endothelial growth factor receptor 2 (VEGFR2), C-X-C Motif Chemokine Ligand 12 (CXCL12), C-X-C chemokine receptor type 4 (CXCR-4), and focal adhesion kinase pathways. In addition, ALC inhibited the activation of nuclear factor kappa-light-chain-enhancer of activated B cells (NF-κB) and intercellular adhesion molecule 1 (ICAM-1) and reduced the adherence of a monocyte cell line to endothelial cells [43].

Functional experiments simulating the pro-tumor development and behavior have shown that ALC inhibits the migration and invasion of four prostate cancer cell lines by reducing cell proliferation. In addition, these experiments revealed that ALC is capable of influencing the crucial functional phases of prostate carcinogenesis, and a number of the implicated molecular mediators were determined [29]. The impact of ALC on two ovarian cancer cell lines (OVCAR-3 and SKOV-3) was evaluated and ALC was found to have no effect on OVCAR-3 cell viability or proliferation and a minor reduction in SKOV-3 cell proliferation [44]. The purpose of this study was to evaluate the possible anticancer outcomes of ALC on human liver cancer cell line HepG2 and colorectal adenocarcinoma cell line HT29, through examining the potential role that ALC could play in preventing cell growth, migration, and gene expression.

## 2. Materials and Methods

### 2.1. Cells Culture and Reagents

The human liver cancer cell line HepG2 and colorectal adenocarcinoma cell line HT-29 were obtained from the American Type Culture Collection (ATCC). Cells were cultured in Dulbecco’s modified Eagle medium (DMEM; Gibco-Invitrogen, Carlsbad, CA, USA) accompanied with 10% fetal bovine serum (FBS), 1% L-glutamine, and 1% penicillin-streptomycin. Cells were maintained at 37 °C in a humidified atmosphere containing 5% CO_2_. ALC was purchased from American International Lab, Inc. (Granada Hills, CA, USA).

### 2.2. Cell Viability Assay

All cell lines were individually seeded in a 96-well plate at 1 × 10^5^ cells/well and incubated overnight at 37 °C in a humidified 5% CO_2_ atmosphere. After 24 h, the medium was replaced with a serum-free medium and incubated for 24 h. Cells were then treated with different dilutions of ALC (0, 0.5, 1,5, 10, 15, 30, and 60 μM) and each incubated for 24, 48, and 72 h. The media were removed after incubation, and the cells were treated with MTT (3-(4,5-dimethylthiazol-2-yl)-2,5-diphenyltetrazolium bromide; Sigma, St. Louis, MO, USA) dissolved at a concentration of 5 mg/mL in PBS. Further, 10 μL of MTT was added to each well, and the cells were incubated at 37 °C for 4 h. The medium was carefully removed, and 100 μL of dimethyl sulfoxide (DMSO; Sigma, St. Louis, MO, USA) was added to each well and mixed. The absorbance of the wells was obtained at 570 nm using a multimode microplate reader (BioTek, Winooski, VT, USA), and the half maximal inhibitory concentration (IC_50_) was calculated for each cell line. Each test was performed six times in triplicate.

### 2.3. Migration Assay

Cells from different cell lines were seeded in a 6-well plate at a density of 5 × 10^5^ cells/well and incubated for 24 h at 37 °C in a humidified 5% CO_2_ atmosphere. Scratch wounds were created in the center of the monolayers of confluent cells using a sterile 200 μL pipette tip [45]. The culture media were removed, the cells were washed with PBS, and each cell line was treated with ALC at their IC_50_. Using an inverted microscope (Leica, Wetzlar, Germany), brightfield images were captured with a 10× objective lens and migration into the wound space was documented at 0, 24, 48, and 72 h.

### 2.4. Detection of Apoptotic DNA

An Apoptosis DNA Ladder Assay Kit (abcam, Cambridge, UK) was used to detect internucleosomal DNA fragmentation in apoptotic cells according to manufacturing protocol. Cells from different cell lines were seeded in 6-well plates at a density of 10 × 10^5^ cells/well for 24 h at 37 °C in a humidified 5% CO_2_ environment. After 24 h, the existing media were replaced with serum-free media and incubated for another 24 h. Each cell line was treated with ALC at their IC_50_ and incubated for 48 h. Untreated cells were used as controls. Cells were trypsinized then pelleted in a 1.5 mL tube, washed with PBS, and pelleted by centrifugation for 5 min at 500× *g*. The supernatant was carefully removed, and cells were lysed with 35 μL of a lysis buffer with gentle pipetting. Then, 5 μL of Enzyme A solution was added to each sample and mixed by gentle vortexing, and cells were incubated at 37 °C for 10 min. Thereafter, 5 μL of Enzyme B solution was added to each sample and incubated at 50 °C for 30 min, followed by the addition of 5 μL of ammonium acetate solution. Then, 50 μL of isopropanol was added, and the solution was mixed well and placed at −20 °C for 10 min. Samples were centrifuged for 10 min, the supernatant was removed, and the DNA pellet was washed with 0.5 mL 70% ethanol and air dried for 10 min. The DNA pellets were dissolved in 30 μL of suspension buffer, and 20 μL from each sample was electrophoresed on 1.5% agarose gel stained with 0.5 μg/mL ethidium bromide.

### 2.5. Fluorescent Microscopy for Evaluating Apoptosis

Cells from different cell lines were seeded in 96-well plates at a density of 0.5 × 10^5^ cells/well for 24 h at 37 °C in a humidified 5% CO_2_ atmosphere. After 24 h, the media were removed and serum-free media were added to each well and incubated for 24 h. Cells were then treated with ALC at their IC_50_ and, after 48 h, the media were removed and the cells were washed twice with PBS, fixed with methanol, and left to air dry at 25 °C for 30 min. The cells were then stained with a 500 nM solution of propidium iodide (PI; Invitrogen) in PBS for 5 min and then rinsed several times in PBS. DAPI (4′,6′-diamidino-2-phenylindole; Life Technologies, Carlsbad, CA, USA) was used to stain cells at a concentration of 1 µg/mL in PBS to detect nuclear morphological changes. The cells were incubated for 5 min in the dark and then rinsed several times with PBS. Cell images were captured using an inverted fluorescent microscope (Leica, Wetzlar, Germany) with appropriate filters for PI and DAPI. ImageJ software was used to overlay the images.

### 2.6. RNA Extraction and Reverse Transcription-Real-Time Polymerase Chain Reaction

Cells were seeded in 6-well plates at a density of 1 × 10^6^ cells/well and incubated for 24 h at 37 °C in a humidified 5% CO_2_ atmosphere. After 24 h, the medium was replaced with a serum-free medium and incubated for another 24 h. Each cell line was treated with ALC at the IC_50_ and incubated for 48 h. TRIzol reagent (Invitrogen) was used to extract total RNA according to the manufacturer’s instructions. The QuantiNova reverse transcription kit (Qiagen, Hilden, Germany) was used to obtain cDNA from extracted RNA according to the manufacturer’s instructions. A 2x PCR master solution (i-Taq, iNtRON Biotechnology, Seoul, Republic of Korea) was used according to the manufacturer’s instructions with the following primers: MMP9 sense: 5′-TTGACAGCGACAAGAAGTGG-3′, antisense: 5′-GCCATTCACGTCGTCCTTAT-3′; VEGF: sense 5′-CCCACTGAGGAGTCCAACAT-3′, antisense: 5′-TTTCTTGCGCTTTCGTTTTT-3′; β-actin: sense 5′-GCTCTTTTCCAGCCTTCCTT-3′, antisense: 5′-GAGCCAGAGCAGTGATCTC-3′. The mRNA expression levels were adjusted to the β-actin expression level and compared with the mRNA expression in the control cells.

### 2.7. Statistical Analysis

GraphPad Prism 9 (GraphPad Software, La Jolla, CA, USA) was utilized for data analysis. The absolute IC_50_ was calculated for each cell line at different time points by converting the concentration to a log concentration value, normalizing the results, and presenting them as percentages followed by nonlinear regression (curve fitting). The two-way ANOVA analysis was used to compare the wound closure % obtained from ImageJ for control vs. treated cells. Unpaired *t*-test analysis was used to compare the percentage of nucleus morphological changes between treated and control cells in each cell line and to compare the expression of each gene in comparison to the control after normalization. Wound closure % was measured using ImageJ version 1.36 (Image J-Fiji, Bethesda, MA, USA) at each time point using Equation (1):

(1) $$\mathrm{wound}\mathrm{closure}\%=\frac{\mathrm{Area}T0-\mathrm{Area}T(24,\;\mathrm{or}\;48\;\mathrm{or}\;72)}{\mathrm{Area}T0}\times 100$$

## 3. Results

### 3.1. Efficacy of ALC on the Viability of HepG2 and HT29 Cell Lines

**Figure 1:**
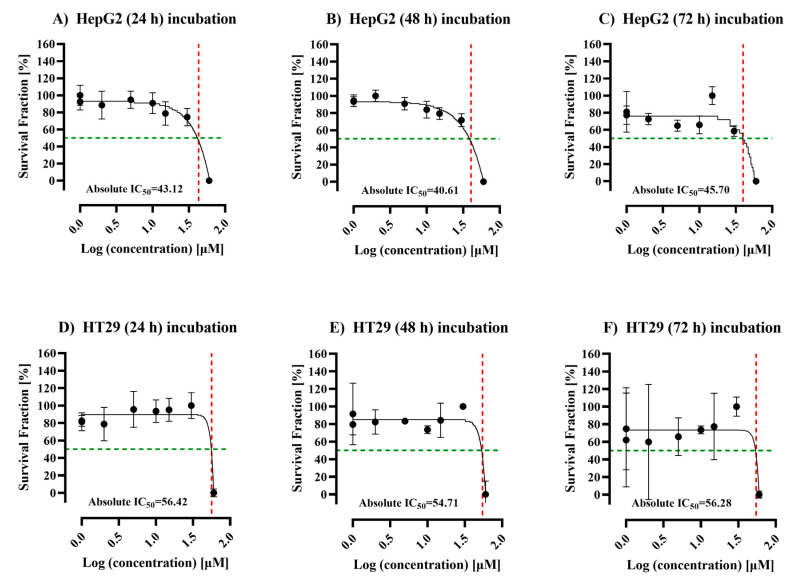
Dose–response curve in HepG2, and HT29 cells after treatment with acetyl-L-carnitine (ALC) at 24, 48, and 72 h. (**A**–**C**) HepG2 cell line, (**D**–**F**) HT29 cell line. Both cell lines were treated with 0, 0.5, 5, 10, 15, 30, and 60 μM/mL at 24, 48, 72 h. The viability was measured at different time points for each cell line. The point where the red dashed line meets the green dashed line is the absolute IC_50_. The concentration was transformed to log concentration and normalized to percentage, then the mean of six repeats was plotted by nonlinear regression. The error bars correspond to standard deviation (SD).

HepG2 and HT29 cells were treated with ALC at different concentrations (0, 0.5, 1,5, 10, 15, 30, and 60 μM/mL) to find the optimum IC_50_. As the concentration of ALC increased, the viability of the cells decreased at the tested time point (Figure 1). The IC_50_ of ALC was determined for each cell line to select the optimal treatment dose and duration for future investigations. The HepG2 cell line showed IC_50_ values of 43.12, 40.61, and 45.70 μM/mL after 24, 48, and 72 h, respectively (Figure 1A–C). The HT29 cell line showed IC_50_ values of 56.42, 54.71, and 56.28 μM/mL after 24, 48, and 72 h, respectively (Figure 1D–F). Based on the results obtained, an IC_50_ of 40.61 μM/mL after 48 h of treatment for the HepG2 cell line and an IC_50_ of 54.71 μM/mL after 48 h of treatment for the HT29 cell line were the optimum conditions for the treatment of both cell lines.

### 3.2. ALC Reduced Migration in HepG2 and HT29 Cell Lines

**Figure 2:**
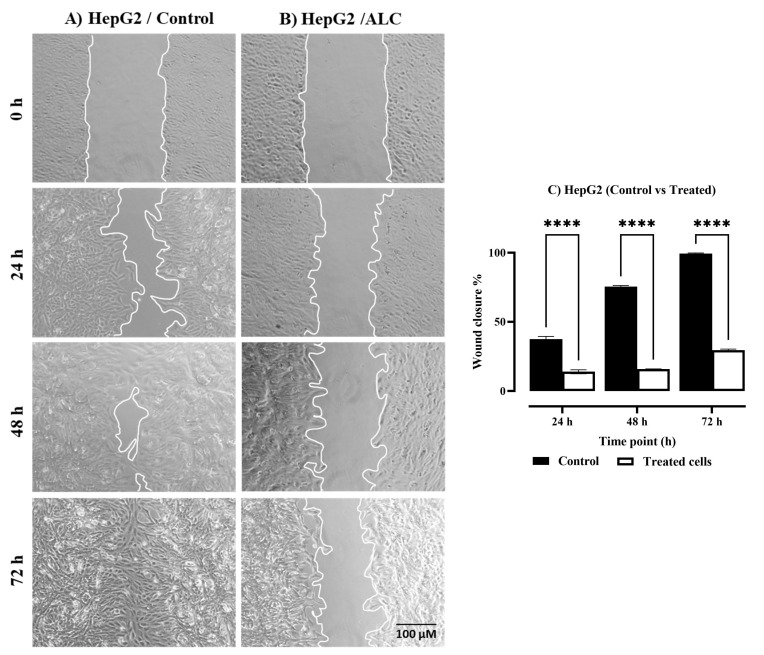
The effect of ALC treatment on wound closure in HepG2 cells at 0, 24, 48, and 72 h. (**A**) Control HepG2 cells, (**B**) ALC-treated HepG2 cells. HepG2 cells were treated with their IC_50_ of ALC, and brightfield images were acquired at 0, 24, 48, and 72 h using an inverted microscope with a 10× objective lens. (**C**) A summary of the wound-healing assay’s findings. ImageJ was used to measure the wound closure percentage. Two-way ANOVA analysis was performed to compare the wound closure percentage for control vs. treated cells. The error bars correspond to SD, **** *p* < 0.0001.

**Figure 3:**
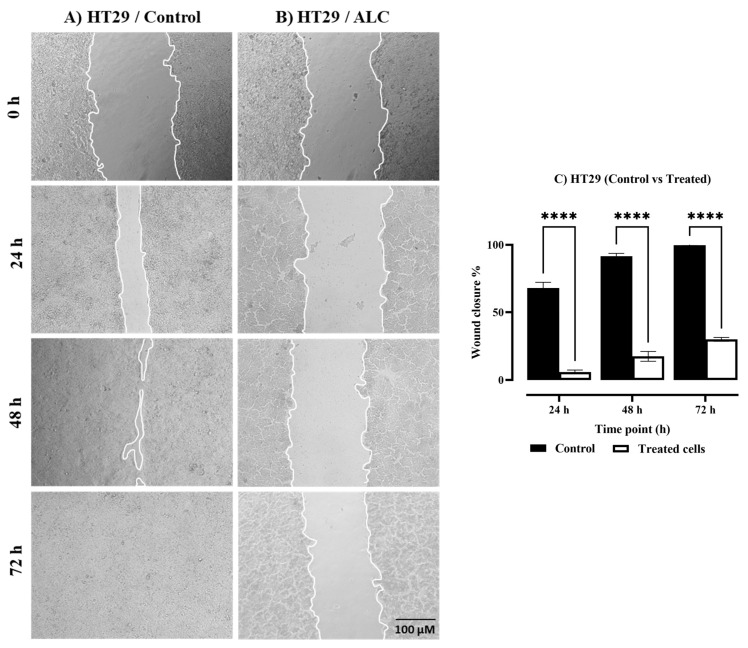
The effect of ALC treatment on wound closure in HT29 cells at 0, 24, 48, and 72 h. (**A**) Control HT29 cells, (**B**) ALC-treated HT29 cells. HT29 cells were treated with their IC_50_ concentrations of ALC, and brightfield images were acquired at 0, 24, 48, and 72 h using an inverted microscope with a 10× objective lens. (**C**) A summary of the wound-healing assay’s findings. ImageJ was used to measure the wound closure percentage. Two-way ANOVA analysis was performed to compare the wound closure percentage for control vs. treated cells. The error bars correspond to SD, **** *p* < 0.0001.

The percentage of wound closure was determined after exposing each cell line to ALC at their IC_50_ at 0, 24, 48, and 72 h. The results in Figure 2A,B showed that treatment with ALC affected the ability of the HepG2 cell line to close the wound after all three tested time points (24, 48, and 72 h) when compared with the control (untreated cells). The HepG2 cell line (Figure 2C) showed a significant reduction when treated with ALC when compared to control cells (*p* < 0.0001). The results in Figure 3A,B clearly show the effect of ALC on the capability of the HT29 cells to migrate and close the wound. Similarly, HT29 cells showed a significant reduction in wound healing compared to control cells (Figure 3C) with *p* < 0.0001.

### 3.3. ALC Influences Nucleus Morphology in HepG2 and HT29 Cell Lines and Induced DNA Fragmentation

**Figure 4:**
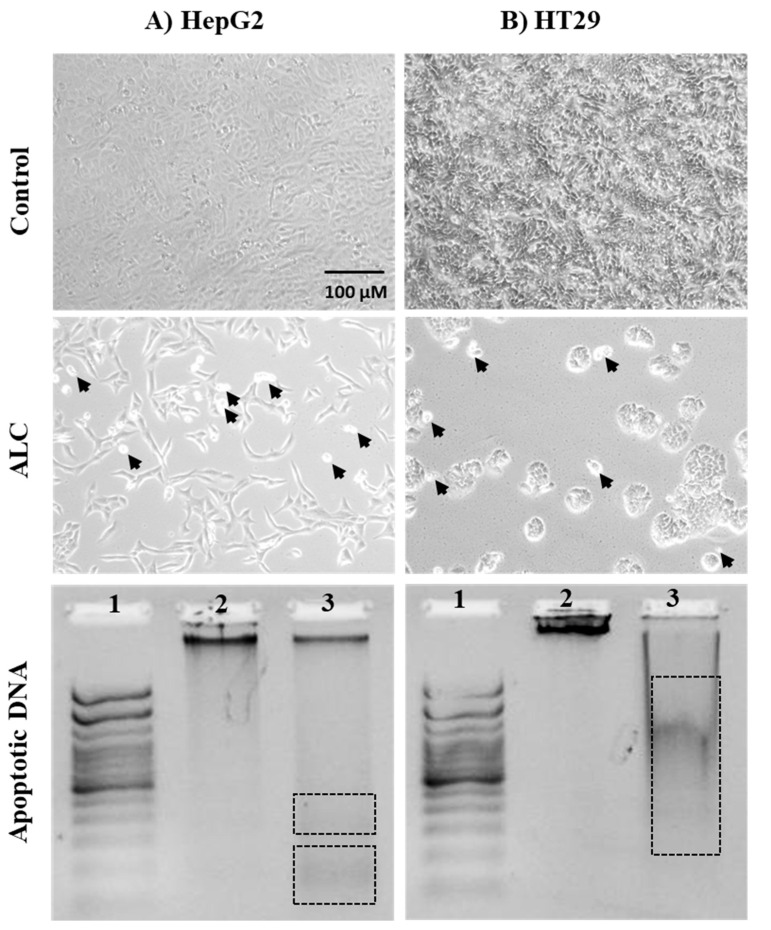
The effect of ALC treatment on cell morphology and DNA fragmentation in HepG2 and HT29 cells. (**A**) HepG2 cells and (**B**) HT29 cells. Cells were treated with their IC_50_ concentrations for 48 h and images of cells were captured under a brightfield microscope. Apoptotic cells are marked by arrows. Gel electrophoresis of apoptotic DNA collected from each cell was performed after treatment with ALC at IC_50_ concentrations for 48 h, (**1**) Kb DNA ladder, (**2**) control cell, (**3**) cell treated with ALC. Apoptotic DNA fragmentation is marked by rectangles.

**Figure 5:**
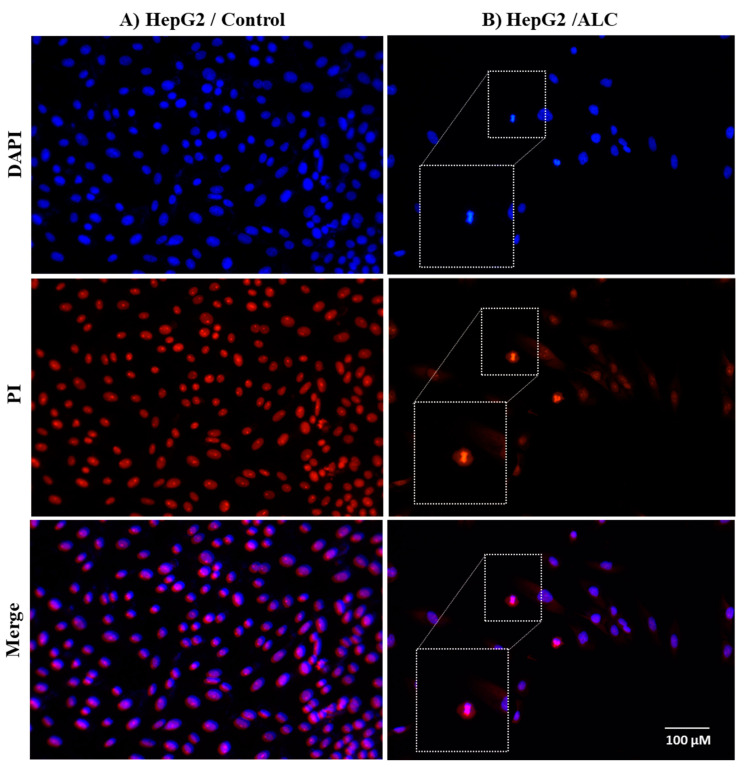
Fluorescent microscopy examination to evaluate apoptotic nucleus morphological changes in HepG2 cells after ALC treatment compared to control. (**A**) control HepG2 cells, (**B**) ALC-treated HepG2 cells. HepG2 cells were treated with their IC_50_ concentration of ALC for 48 h and stained with DAPI and propidium iodide (PI). Brightfield images were acquired under fluorescence microscopy with appropriate filters for PI and DAPI. ImageJ was used to overlay images. Rectangles show some cells after being magnified to illustrate the apoptotic effect of ALC on the nucleus.

**Figure 6:**
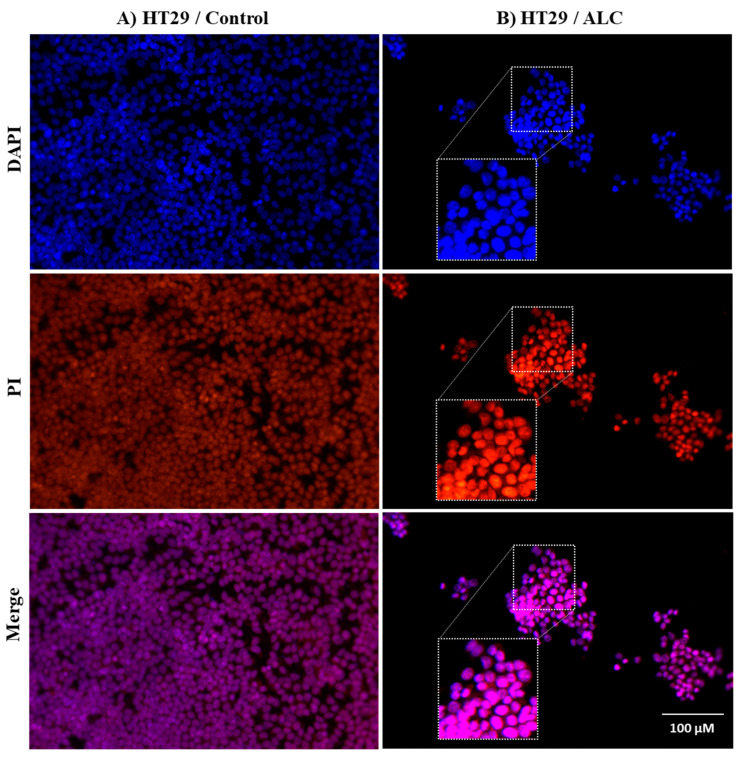
Fluorescent microscopy examination to evaluate apoptotic nucleus morphological changes in HT29 cells after ALC treatment compared to control. (**A**) control HT29 cell line, (**B**) ALC-treated HT29 cell line. HT29 cell lines were treated with their IC_50_ concentration of ALC for 48 h and stained with DAPI and PI. Images were acquired under fluorescence microscopy with appropriate filters for PI and DAPI. ImageJ was used to overlay images. Rectangles show some cells after being magnified to illustrate the apoptotic effect of ALC on the nucleus.

**Figure 7:**
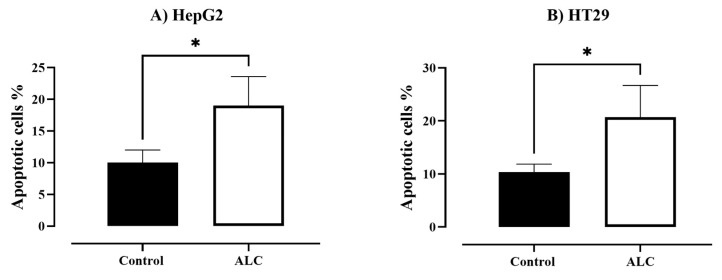
Apoptotic cells (%) under fluorescent microscopy in HepG2 and HT29 cells after ALC treatment compared to control. (**A**) HpG2 cells, (**B**) HT29 cells. The proportion of apoptotic cells in each cell line after the treatments were determined using inverted fluorescence microscopy. An unpaired *t*-test analysis was used to compare the apoptotic % between treated and control in each cell line. Data expressed as means ± SE of three experiments * *p* < 0.05 compared to control.

To investigate the effect of ALC on HepG2 and HT29 cell lines, changes in nuclear morphology were observed under both brightfield and fluorescence microscope. Both cell lines showed normal morphology with no treatment (control), while apoptotic cells were detected in both cells treated with ALC (Figure 4). Further changes to the cells include decrease in size, increase in cell density, and the chromatin condenses and migrates to the edges of the nucleus. Both cell lines showed DNA fragmentation after treatment with ALC; based on gel electrophoresis results, it is most likely that HT29 treated cells showed more DNA fragmentation than did HepG2 cells. The fluorescence images obtained after treating HepG2 cells with ALC support these findings, showing that treated cells were more likely to undergo apoptosis than untreated control cells (Figure 5). The findings for the HT29 cell line when exposed to ALC were similar (Figure 6), which may indicate that ALC has a nuclear morphological effect in both cell lines. In fact, the nucleus morphological change significantly increased (*p* < 0.05) in both cell lines after treatment with ALC, as shown in Figure 7A,B.

### 3.4. ALC Downregulates the Expression Level of Matrix Metallopeptidase 9 (MMP9) and Vascular Endothelial Growth Factor (VEGF) in HepG2 and HT29 Cell Lines

**Figure 8:**
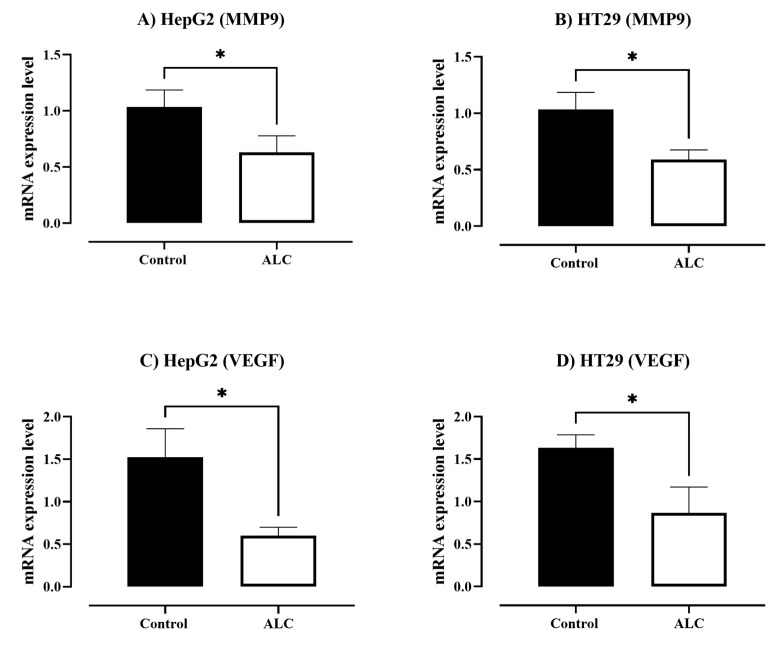
The mRNA expression of MMPs and VEGF in HepG2 and HT29 cells after ALC treatment. (**A**) MMPs mRNA expression levels in HepG2, (**B**) MMPs mRNA expression levels in HT29, (**C**) VEGF mRNA expression levels in HepG2, and (**D**) VEGF mRNA expression levels in HT29. The expression levels of all genes were normalized to that of the β-actin gene. An unpaired *t-*test analysis was used to compare the expression of each gene compared to the control. Data expressed as means ± SE of three experiments. * *p* < 0.05 compared to control.

To determine the effect of ALC on HepG2 and HT29 cells, the mRNA expression levels of MMP9 and VEGF were determined. The results obtained in Figure 8A,C show a significant decrease in mRNA expression levels of MMPs and VEGF in both cell lines treated with ALC (*p* < 0.05) compared with the control.

## 4. Discussion

Although chemotherapy is currently one of the most important techniques used to treat cancer [46], the side effects are a serious disadvantage [47]. Chemoprevention of cancer with either natural or synthetic drugs is a potential method for reducing disease prevalence [48], and consequently, in the past few years, a significant amount of research has been conducted on the preventive and therapeutic abilities of several natural compounds and nutritional supplements against certain types of cancer [49]. ALC is an acetylated derivative of carnitine that plays a crucial function in intermediate metabolism in most tissues [50,51]. The anti-inflammatory and antioxidant effects of ALC have been proven in several studies [35,52,53], as well as its potent anticancer properties [29]. Some studies have demonstrated that ALC may play a significant role in some DNA modifications, such as histone acetylation, which may result in alterations in gene expression [54,55]. In the current study, two cancer cell lines, HepG2 and HT29, were investigated in vitro to determine the therapeutic effect of ALC on their cell viability, migration, morphology, and gene expression. The findings of this study demonstrate the significant anticancer action of ALC on HepG2 and HT29 cancer cell lines, consistent with previous research. In this study, we found that the IC_50_ of ALC on HepG2 cells was 43.12, 40.61, and 45.70 µM after 24, 48, and 72 h of treatment, respectively, whereas the IC_50_ of ALC on HT29 cell was 56.42, 54.71, and 56.28 µM after 24, 48, and 72 h of treatment, respectively. A previous in vitro investigation to elucidate the effects of ALC at concentrations of 0, 1, 10, and 100 μM on the proliferation of OVCAR-3 and SKOV-3 ovarian cancer lines using flow cytometry showed that there was a minor, but significant, reduction in the proliferation of the ovarian cancer cell line SKOV-3 when exposed to 10 µM and 100 µM ALC [44]. SW480 human colon cancer cell lines treated with 2 or 3 mM butyrate, with or without carnitine or ALC at a concentration of 5 mM for 48 h, caused a significant increase in the death of SW480 cells [42].

For prostate cancer cell lines PC3, DU145, LNCaP, and 22Rv1, ALC concentrations of 1 and 10 mM were determined to be effective in preventing cellular proliferation [45]. Human umbilical vein endothelial cells (HUVEC) were exposed to 1 or 10 mM ALC for 24 h and the results showed that ALC had a dose-dependent effect on HUVEC proliferation [43]. The effect of ALC on the longevity and proliferation of other human cell lines, including MRC5 and peripheral blood mononuclear cells collected from healthy people, has been previously investigated. At the maximum dose (10 mM), ALC had no influence on the proliferation of healthy cells [43].

Cell migration is an important approach, in which cells must be able to move and reach their correct place within a particular environment in order to carry out their activity [56]. In this study, we found that ALC reduces migration in both cell lines significantly when compared to untreated cells. This is in line with previous research which showed that ALC prevents prostate cancer cell lines from adhering, migrating, and invading [29].

In this study, we examined the cells under a microscope to detect morphological changes that could lead to apoptosis and cell death, and performed a DNA fragmentation assay. The results obtained showed that ALC induced apoptosis when compared to untreated cells. Other studies have evaluated the effect of carnitines on cancer cells in vitro and found that both apoptosis and DNA fragmentation are promoted by carnitine in malignant cells, in line with the present study finding [23,57,58].

Another study evaluated the anti-angiogenic and chemopreventive effects of ALC on four types of prostate cancer cell lines, and found that ALC significantly decreased cell division, promoted apoptosis, and inhibited the synthesis of pro-inflammatory cytokines and chemokines. They also found that ALC reduces cell migration, adhesion, and invasion properties through the downregulation of MMP-9, C-X-C chemokine receptor type 4 (CXCR-4), and the chemokine (C-C motif) ligand 12 (CCL12) pathway, which also induces the inhibition of the angiogenesis pathway via VEGF and C-X-C motif chemokine ligand 8 (CXCL8) [29]. Research in mouse models of cancer cachexia found that the anti-inflammatory cytokines TNFα and IL-6 were significantly inhibited, and the biochemical parameters were enhanced after the administration of oral L-carnitine. They concluded that L-carnitine, in conjunction with the PPAR-γ signaling pathway, exhibits beneficial impacts on cancer cachexia [59]. The impact of L-carnitine on the activities and mRNA expression levels of carnitine palmitoyltransferase (CPT) I and II in the livers of mice with cachectic cancer had been previously evaluated, with findings showing that significant elevations in the expression of CPT I and II were associated with a significant reduction in serum levels of TNF-α and IL-6 [60]. The ability of L-carnitine and ALC with or without the combination of curcumin to prevent cancer development via the 1,2-dimethylhydrazine-stimulated colon tumor mouse model was evaluated for 20 weeks and it was found that both L-carnitine and ALC inhibited the formation of neoplastic lesions as effectively as curcumin alone, if not more so [61]. The combination of palmitoylcarnitine and carnitine promotes oxidative stress and apoptosis in HT29 cells by boosting the efficiency of mitochondrial respiration [57]. Another study found that L-carnitine or palmitoylcarnitine alone could boost caspase-3 and DNA fragmentation, but when administered simultaneously apoptosis was induced [58]. The combination of butyrate and carnitine inhibited the proliferation of human colon cancer Caco-2 cells and induced apoptosis by upregulating proapoptotic proteins (BAX and BAK) and downregulating anti-apoptotic proteins (BCL); however, treatment with carnitine alone did not affect the expression of BAX and BAK, despite the fact that apoptotic effects were observed [23]. The mechanisms behind the antitumor effect of carnitine and ALC on the response of SW480 colon cancer cells to butyrate were investigated, and the combination of butyrate and ALC was found to enhance the death rate of cells. Additionally, carnitine and ALC boosted apoptosis induction, with ALC alone generating a 20% reduction in p21. There was no impact of carnitine or ALC on BCLXL expression. The conclusion was that butyrate and ALC exhibit high antitumor effects and inhibit the viability of colon cancer SW480 cells [42]. In contrast, however, another study reported that L-carnitine administration had no cytotoxic effect on HepG2 cells [62]. Our data showed that, at the molecular level, the incubation of HepG2 and HT29 cells with ALC could reduce MMP expression, with MMPs likely being involved in the invasive properties of cancer cells [63,64]. Our data show similar results to previous research, showing that ALC downregulates the expression of MMP9, which likely suppresses the in vitro hallmarks of tumor progression by inhibiting adhesion, migration, and invasion of four prostate cell lines [29]. In this study, ALC significantly downregulated the expression level of VEGF. VEGF has been shown to perform a crucial function in tumor angiogenesis [64,65,66,67]. The effect of ALC as an “angiopreventive” was examined previously in vivo and in vitro at the molecular level, and it was found that ALC is capable of reducing the expression of several pathways, including VEGF pathways. ALC inhibited the stimulation of NF-B and ICAM-1, hence decreasing the adherence of a monocyte cell line to endothelial cells, leading to the conclusion that ALC has anti-angiogenic and anti-inflammatory characteristics. This could make cancer angioprevention possible [43].

The main limitation of the present study is that multiple diverse colorectal cancer and liver cancer cell lines were not used. However, future research could include the validation of the effect of ALC on additional colorectal cancer and liver cancer cell lines.

In conclusion, ALC has antitumor activity against HepG2 and HT29 cell lines. Based on the obtained results, it is most likely that the antitumor effect of ALC is mediated by decreasing adhesion, migration, and invasion. The results of the present study suggest that ALC might be used as a possible chemopreventive supplement for liver and colon cancer. Further in vivo and in vitro investigations are required to evaluate the efficacy of ALC as a chemopreventive drug for clinical use.

## Data Availability

No additional data were created.
